# New grasslands promote pollination but not biological pest control in nearby arable fields in the short term

**DOI:** 10.1007/s11829-023-10034-5

**Published:** 2024-01-31

**Authors:** Manuela Bürgler, Raja Imran Hussain, Bea Maas, Ronnie Walcher, Dominik Rabl, Bernhard Krautzer, Dietmar Moser, Thomas Frank

**Affiliations:** 1 Institute of Zoology, Department of Integrative Biology and Biodiversity Research (DIBB), University of Natural Resources and Life Sciences, Gregor-Mendel-Straße 33, 1180 Vienna, Austria; 2 Biodiversity Dynamics and Conservation, Department of Botany and Biodiversity Research, University of Vienna, Rennweg 14, 1030 Vienna, Austria; 3 Institute for Plant Production and Cultural Landscape, Department for Ecological Restoration, Agricultural Research and Education Centre Raumberg-Gumpenstein, Altirdning 11, 8952 Irdning-Donnersbachtal, Austria; 4 Applied Ecology Unit, School of Natural Sciences, University of Galway, Galway, Ireland

**Keywords:** Ecosystem service, Semi-natural grassland, Distance effect, Pollinator, Predatory arthropod

## Abstract

Agricultural intensification is a major threat to farmland biodiversity and associated ecosystem services. Semi-natural habitats are integral to the preservation of farmland biodiversity and ecosystem services, however, the extent in which they contribute to specific services is largely unclear. We studied predation rates of ground-dwelling predatory arthropods, and pollination success within old permanent grasslands, newly established grasslands and arable fields near and far from new grasslands. We evaluated whether grassland restoration can enhance pollination and biological control in crop fields. For this purpose, we established new grassland strips within cereal fields, which directly bordered existing permanent grasslands. We evaluated if the distance to these old and new grasslands affects the delivery of the two ecosystem services within crop fields. We found significantly higher seed numbers and seed weight in sentinel plants placed in old grasslands, new grasslands and nearby arable fields compared to distant arable fields. We also found significantly decreasing seed numbers and seed weight in sentinel plants placed in distant arable fields with increasing distance from old grasslands, while pollination success was not affected by distance in nearby arable fields. Contrary, we did not find any significant effects of new grasslands on biological control. Our study showed that 3 years after establishing grasslands arable fields benefited from the proximity of flower-rich new grasslands through increased pollination success though not regarding biological control. This indicates that, on a short term, establishing new grasslands can support beneficial arthropods in providing ecosystem services such as pollination. Predators, in contrast, might take longer to establish effective populations that denote higher predation rates. Our study provides a baseline for future long-term studies to better evaluate pollination and pest control patterns within arable fields.

**Supplementary Information:**

The online version contains supplementary material available at 10.1007/s11829-023-10034-5.

## Introduction

Biodiversity loss and environmental degradation through land-use intensification and land-use change disrupts ecosystem services of beneficial arthropods such as pollination and biological pest control, threatening sustainability of food production (Tscharntke et al. 2012). In Europe, several agri-environmental schemes have been initiated (e.g., Kleijn and Sutherland 2003; Aviron et al. 2009) to address the alarming biodiversity loss and the associated loss of ecosystem services in agroecosystems (e.g., Méndez-Rojas et al. 2021; Raven and Wagner 2021).

Functionality and efficacy of ecosystem services like pollination or biological pest control rely on the presence of beneficial insect species. Pest emergence can be effectively reduced by the presence of ground-dwelling predators in agricultural fields (Zaller et al. 2009). Aside from abundance, also species richness of pollinators and natural enemies plays a major role in ecosystem service efficacy. Up to half of all negative impacts of landscape simplification on ecosystem services and associated crop yield losses can be caused by losses in richness of service-providing beneficial organisms (Dainese et al. 2019).

Interspersed semi-natural habitats can indeed enhance species diversity and abundance of pollinators and epigeic predators on arable land (Albrecht et al. 2007; Haaland et al. 2011; Arathi et al. 2019). Acreage of semi-natural habitats and spill-over effects positively influence communities of predatory arthropods and natural pest control within agricultural fields (Boetzl et al. 2020a). Unfortunately, due to tremendous declines in EU subsidies for set-aside farmland since 2008, these semi-natural habitats disappeared rapidly from European agroecosystems (Pe’er et al. 2014).

Especially older fallows seem to promote functionally more diverse communities of natural enemies, thus, positively affecting adjacent arable fields (Feng et al. 2021). Therefore, maintaining and restoring permanent grasslands is an effective measure to foster functional diversity in predatory arthropod communities (Maas et al. 2021). In a previous study, however, we could show that epigeic predatory arthropods react much slower to restored grasslands compared to pollinators (Hussain et al. 2021).

A large number of studies have already addressed the issue of the age of semi-natural habitats and their impact on insect communities. However, results are not always clear-cut. In a recent study, butterfly species were recorded inside wildflower strips over 10 years. Their population showed an increase of 82%, which the authors attribute to the strips temporal continuity and availability of key host plants, which offered generalist and specialist species alike to find resources for food and reproduction (Kolkman et al. 2022). They highlight the importance of long-term application of wildflower strips as an instrument to contribute to butterfly conservation. Carvell et al. (2004) could show that the attractiveness of certain margin types for bumblebees (*Bombus* spp., Hymenoptera: Apidae) varied between years as a result of the changing availability of different food resources.

Regarding ground-dwelling predatory arthropods, often there is little difference between wildflower strips and other field margin types because these species are typically less dependent on floral resources, but the age of the field margin and the time of the year affect abundance. In Frank et al. (2012), beetle abundance (Coleoptera) decreased significantly with the age of wildflower areas, showing that wildflower areas did not promote larger populations with increasing habitat age. However, diversity and evenness increased significantly with habitat age. In a review, Haaland et al. (2011) showed the wide diversity of results from different studies on the importance of sown wildflower strips for insect conservation and the role of succession and age of wildflower strips and similar landscape structures. Several studies observed changes in diversity and abundance of different species over the years, especially where there is little management, vegetation structure and where the flowering plant community changes with succession. In several cases, studies showed mixed results. For example, the effect of age of wildflower areas on density of carabid beetles (Coleoptera: Carabidae) seemed to be highly dependent on individual species (Frank et al. 2007). However, many studies show a definite increase in abundance with increasing age of semi-natural habitats, e.g., Luka et al. (2006) for true bugs (Hemiptera: Heteroptera) and cicadas (Hemiptera: Auchenorrhyncha), and Jacot et al. (2007) for butterflies (Rhopalocera) and grasshoppers (Orthoptera). Age also seems to affect the quality of wildflower areas as overwintering habitat for beetles, which differently affected species richness and abundance of overwintering staphylinids (Coleoptera: Staphylinidae) and carabids (Frank and Reichhart 2004). Changes in community structure in wildflower areas over the years could also be observed for bugs. While species richness and abundance did not differ between wildflower areas of different age, the number of predatory true bugs increased and communities became more dissimilar over the years (Frank and Künzle 2006). Haaland et al. (2011) concluded that leaving wildflower strips in place for several years and ensuring that strips of different age are available provides the greatest overall benefit for insect biodiversity.

In this study we investigated the short-term effects of newly established grasslands on ecosystem services. For 3 years, we observed pollination success of sentinel plants and biological pest control in new grasslands (NG), already existing older meadows (OG), in arable fields near to NG (CN) and arable fields far away (CF) and at different distances from OG in interaction with distance to NG.

We addressed the following questions:

(i) Do NG significantly enhance ecosystem service efficacy of biological pest control and pollination in CN compared to CF in a short term?
(ii) Is there a distinct decay in biological pest control and pollination with increasing distance from OG within NG, CN and CF?

The OG, traditional for the Wienerwald study region, represent the semi-natural reference habitat and we designed the NG specifically to mimic the OG by using adequate seed mixtures. Our hypotheses were (i) higher predation and pollination success within OG compared to NG because of supposed higher activity density and species richness of beneficial arthropods in OG; (ii) higher predation and pollination success in CN compared to CF due to spill-over effects from NG. Furthermore, we expected (iii) a distinct decay in predation and pollination success with increasing distance from OG in CF but a less pronounced effect in CN because of spill-over from NG. In NG we expected no or minimal distance decay since they provided both permanent, low-disturbance habitats for predatory arthropods and a constant food supply for pollinators.

## Materials and methods

### Research area and study sites

**Fig. 1:**
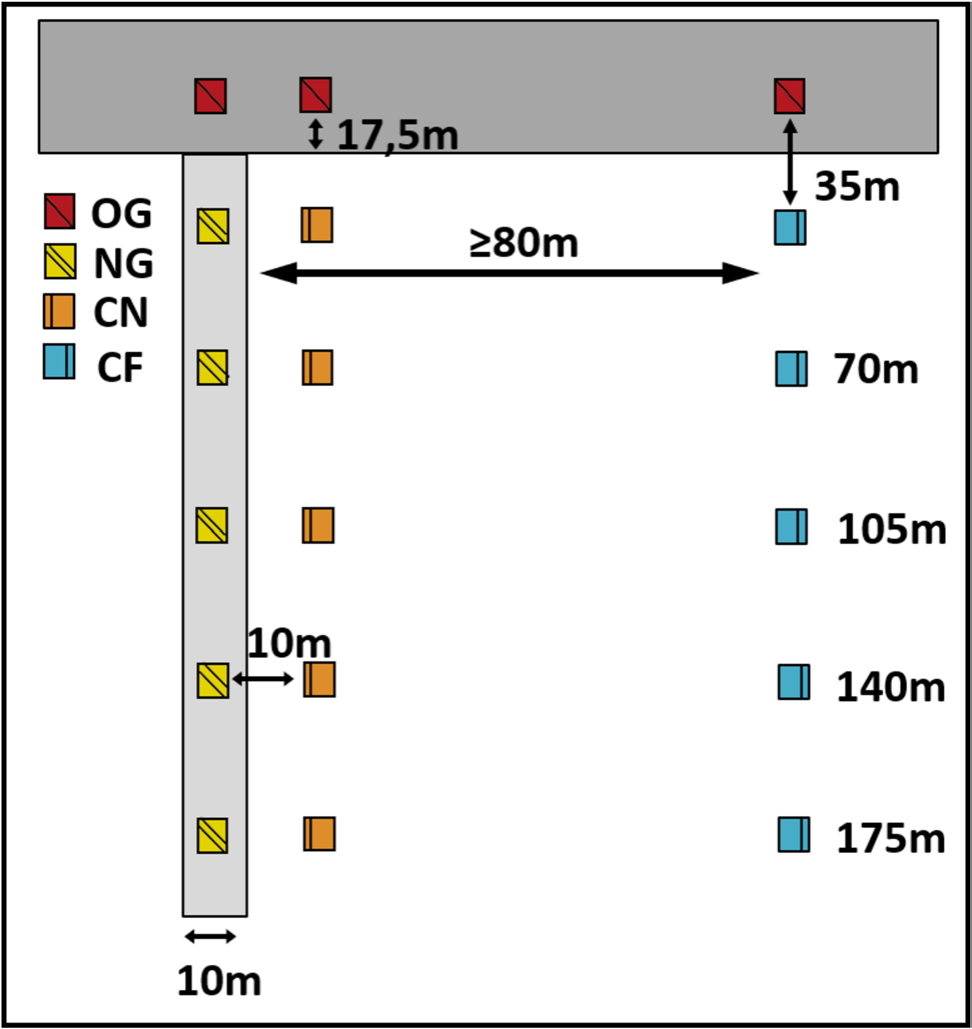
Positioning of sampling plots along three transects (*NG* new grassland, *CN* cereal near NG, *CF* cereal far from NG) with increasing distance from the old grassland (*OG*). The first plot of each transect is situated within OG

The study was conducted between April and June 2017–2019. Five study sites were located in Ollern (48° 16′ 02.5″ N 16° 05′ 07.9″ E) and Elsbach (48° 15′ 08.3″ N 16° 02′ 56.9″ E) (Lower Austria, Austria, mean annual air temperature: 9.9 °C, mean annual precipitation: 673 mm). Despite intensive agricultural management, the study region is quite heterogeneously structured. In August 2016, five new grassland strips (NG, 10 m wide and 180 m long) were sown in winter cereal fields adjacent to extensively managed meadows (OG) (Fig. 1). While cereals are not insect-pollinated crops, we used pollination within cereal fields as a model for ecosystem performance relative to distance of newly established semi-natural habitats. For the establishment of NG we used a seed mixture specifically composed to mimic OG. To this end, we analysed plant relevés from meadows within and near the study region (28 samples of *Filipendulo-Arrhenatheretum* and 54 samples of *Ranunculobulbosi-Arrhenatheretum* grasslands; Hülber et al. 2017). From the 50 most abundant plant species, occurring in at least 25% of the samples, we selected 41 plant species in accordance to seed material availability (Supplementary Information Table SI.1). The total seed weight of the resulting composition consisted of 34.1% grasses, 14.6% legumes and 51.3% other herbs (see Hussain et al. 2021 for further details). These NG were mown once a year in late summer. The cereal arable fields adjacent to the grasslands were managed conventionally by farmers.

Observations were conducted in OG and along transects within NG, CN and CF (Fig. 1). Sampling started always with the first plots in the adjacent OG and continued with five more plots spaced along the transects into NG, CN, and CF, respectively.

Each sampling plot was set 35 m apart from each other and from the corresponding plot in the OG. The last plots of each transect, which was placed farthest away from OG, were either in the centre of an arable field or, in one case, bordered a road followed by another arable field. No other meadows were in close proximity to the sampling plots. With five replicates for each of the three habitats (NG, CN, and CF) a total of 90 sampling plots was used for ecosystem service evaluation per sampling run (3 habitat types × 5 sites × 6 plots per transect = 90 sampling plots).

### Biological pest control

For estimation of biological pest control efficacy, we observed predation rate of epigeic predatory arthropods on adult *Drosophila melanogaster* flies (Diptera: Drosophilidae). Using *Drosophila* flies as prey has proven to be a reliable and cost-effective method for assessing predation rates of ground-dwelling predators (Zaller et al. 2009; Boetzl et al. 2020b). Sampling was done three times between April and May in three consecutive years. On rectangular pieces of cardboard (6 × 7 cm) 30 flies were glued with fish glue (“prey cards”) and pinned to the ground with large metal nails. A thin plastic sheet prevented soil moisture from soaking the cardboard. To prevent access of rodents and birds we put metal mesh cages (8 mm mesh size) on top of each card. On each sampling plot two prey cards were positioned 4 m apart along the transect line with the plot centre in the middle (2 m distance to each prey card). In total, 180 prey cards were used per sampling run (90 sampling plots × 2 two prey cards per plot). After an exposure of 24 h in the field, we recorded the number of consumed flies on each card.

### Pollination success

For evaluation of pollination success, we measured seed number and seed weight in the insect pollinated, largely self-incompatible, perennial herb *Hypochaeris radicata* (Asterales: Asteraceae; Albrecht et al. 2007; Pico et al. 2004). The test plants were grown in greenhouses in the Botanical Garden of the University of Vienna and brought to the field in May when they started flowering. Previously opened buds were removed before transport to exclude any prior pollination. Pollination experiments were performed once a year between 2017 and 2019. One pot with three plants each was exposed for 1 week on every sampling plot. After 1 week in the field the plants were brought back to the Botanical Garden and covered in gauze to prevent further pollination. When seed development was complete, seeds were removed for counting and weighing in the laboratory. Test plants brought to the field varied in number of unopened buds, but featured at least three buds per plant. To account for this in the pollination success assessment, we selected the flower head (capitulum) with the highest number of seeds per plot. Seeds of this one flower head were then counted (maximum number of seeds) and weighed and mean seed weight was calculated.

In 2017, high pest activity and subsequent mortality rate of test plants increased the loss rate in our data to such an amount that we decided to remove them from statistical analysis.

### Statistical analysis

Statistical analyses were done using the software R (R Core Team 2018, version 4.1.2). For pollination data, we did only one observation per year (2018 and 2019, s.o.) and used the maximum number of ripe seeds per flower head per plot (i.e., we counted the seeds in all flower heads per plot separately and used the number of the flower head with the maximum seeds as our response). The second response for pollination was the mean seed weight across all harvested seeds per plot. Therefor we used 15 (transects) × 6 (plots) × 2 (years) = 180 observations for pollination.

For analysis of predation data (i.e., number of eaten flies) we used data from 3 years (2017–2019). As response we used the number of eaten larvae pooled from three runs per year, and two cards (30 flies per card) per plot. Therefore, the sample size summed up to 15 (transects) × 6 (plots) × 3 (years) = 270.

For all three response variables (maximum number of seeds, mean seed weight and number of eaten flies) we tested for differences between habitat types (NG, OG, CN and CF) and we tested the response to distance from semi-natural habitats (OG) by using mixed-effect models. All models included sampling plot nested in transect as random effects for intercept to account for our sampling design. When testing for differences between habitat types, the habitat type (NG, OG, CN and CF) and the year of observation entered as additive fixed effects. When testing for the response to the distance from semi-natural habitats (OG) we excluded the OG plots and used the interaction between habitat type and distances and the year of observation as additive fixed effect predictors.

The maximum number of seeds and eaten flies were count data, hence we used Poisson family generalized linear mixed models with a log-link (glmer function R package lme4, Bates et al. 2015). Since these models showed indication of overdispersion (i.e., residual deviance was larger than the degree of freedom, tested with dispersion_glmer function from the blmeco R package, Korner-Nievergelt et al 2015), we included an additional observation-level random effect to account for the overdispersion (Harrison 2014). The mean seed weight were continuous data (measured in decimal gram) and were analysed with linear mixed models (lmer function from R package lme4, Bates et al. 2015). Shapiro–Wilk test was used to check for normal distribution of the data. Post hoc pairwise comparisons of habitat types were calculated with emmeans function from the emmeans package in R (Lenth 2023).

## Results

### Biological pest control

**Fig. 2:**
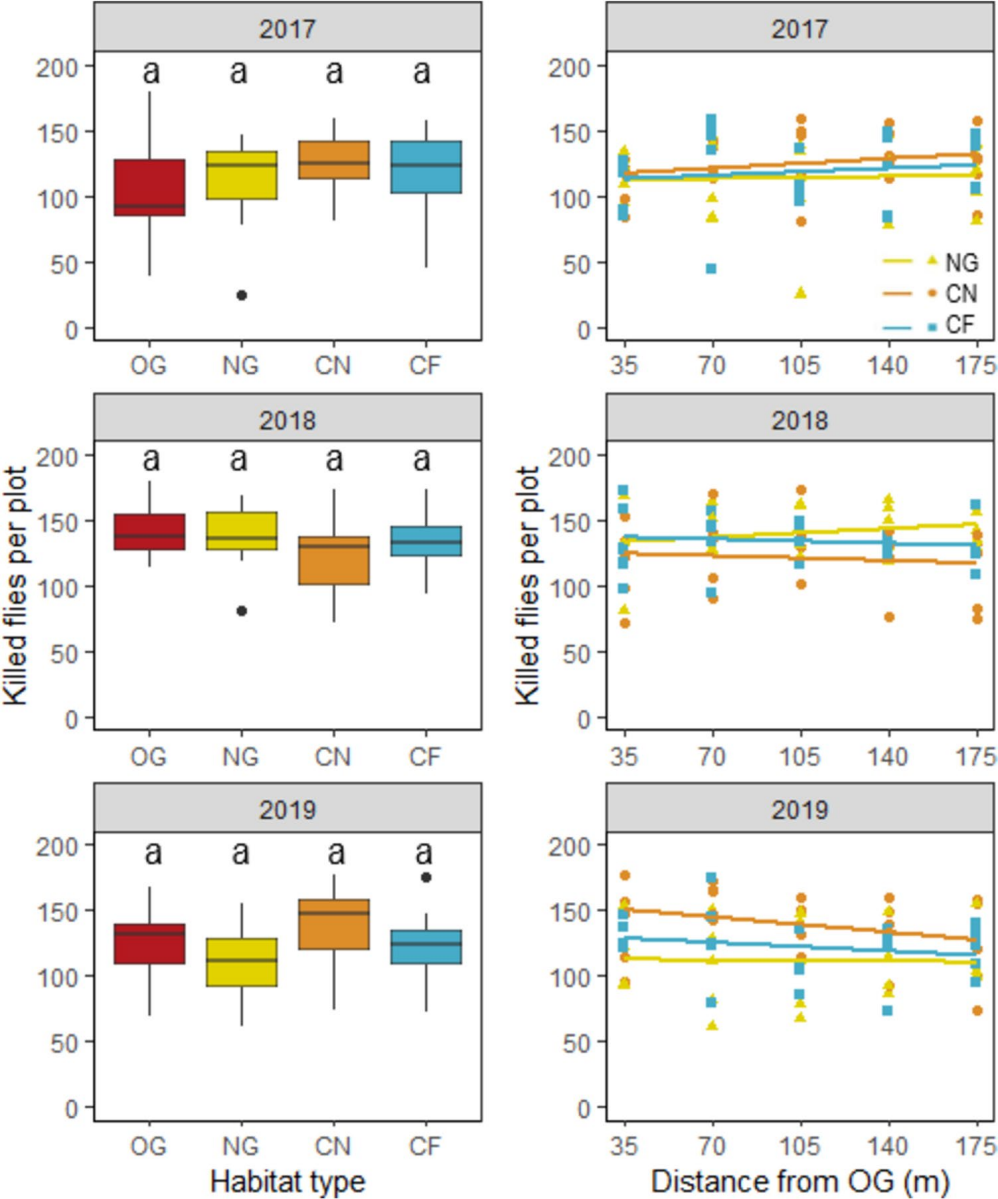
Biological pest control—Killed (consumed) flies per study plot in the four habitat types (*OG* old grassland, *NG* new grassland, *CN* Cereal near NG, *CF* cereal far from NG) in 2017, 2018 and 2019 (left) and along the transects in NG, CN and CF with increasing distance from OG (right)

The individual years differ greatly and the highest predation rates were recorded in 2018. However, there were no significant differences between OG, NG, CN and CF over the years. Additionally, we did not detect any distance effects over the 3 years in any of the study transects (Fig. 2; Table 1).

**Table 1:** Summary of GLMM testing for differences in number of killed flies between the four habitat types (*OG* old grassland, *NG* new grassland, *CN* cereal near, *CF* cereal far) and at different distances from OG within NG, CN and CF

Biological pest control—habitat types					
	Estimate	SE	*z* value	*p* value	
Intercept	4,699	0,040	117,641	< 0,001	***
OG	0,005	0,044	0,116	0,908	
CN	0,058	0,037	1,559	0,119	
CF	0,038	0,045	0,833	0,405	
Y2018	0,150	0,034	4,385	< 0,001	***
Y2019	0,074	0,034	2,154	0,031	*

**Multicomparison**					
Contrast	Estimate	SE	*z* ratio	*p* value	
NG—CF	− 0,04	0,05	− 0,83	0,839	
NG—CN	− 0,06	0,04	− 1,56	0,402	
NG—OG	− 0,01	0,04	− 0,12	0,999	
CF—CN	− 0,02	0,05	− 0,46	0,968	
CF—OG	0,03	0,05	0,70	0,897	
CN—OG	0,05	0,04	1,21	0,621	

Biological pest control—distance effect					
	Estimate	SE	*z* value	*p* value	
(Intercept)	4,7650	0,0535	89,1490	< 0,001	***
Years of observation	0,0232	0,0186	1,2460	0,213	
NG:Distance to OG	− 0,0002	0,0004	− 0,5250	0,600	
CN:Distance to OG	0,0001	0,0004	0,3920	0,695	
CF:Distance to OG	− 0,0001	0,0004	− 0,1690	0,866	

**Multicomparison**					
Contrast	Estimate	SE	*z* ratio	*p* value	
NG:Distance to OG—CF:Distance to OG	− 0,01	0,04	− 0,35	0,934	
NG:Distance to OG—CN:Distance to OG	− 0,03	0,03	− 1,04	0,553	
CF:Distance to OG—CN:Distance to OG	− 0,02	0,04	− 0,57	0,835	

Estimates of respective variable in the final model, standard errors (SE), *z*-values (z), *z*-ratios and *p*-values (*p*) with significant effects marked as . *p* < 0.1, *p < 0.05, **p < 0.01, ***p < 0.001

### Pollination success

**Fig. 3:**
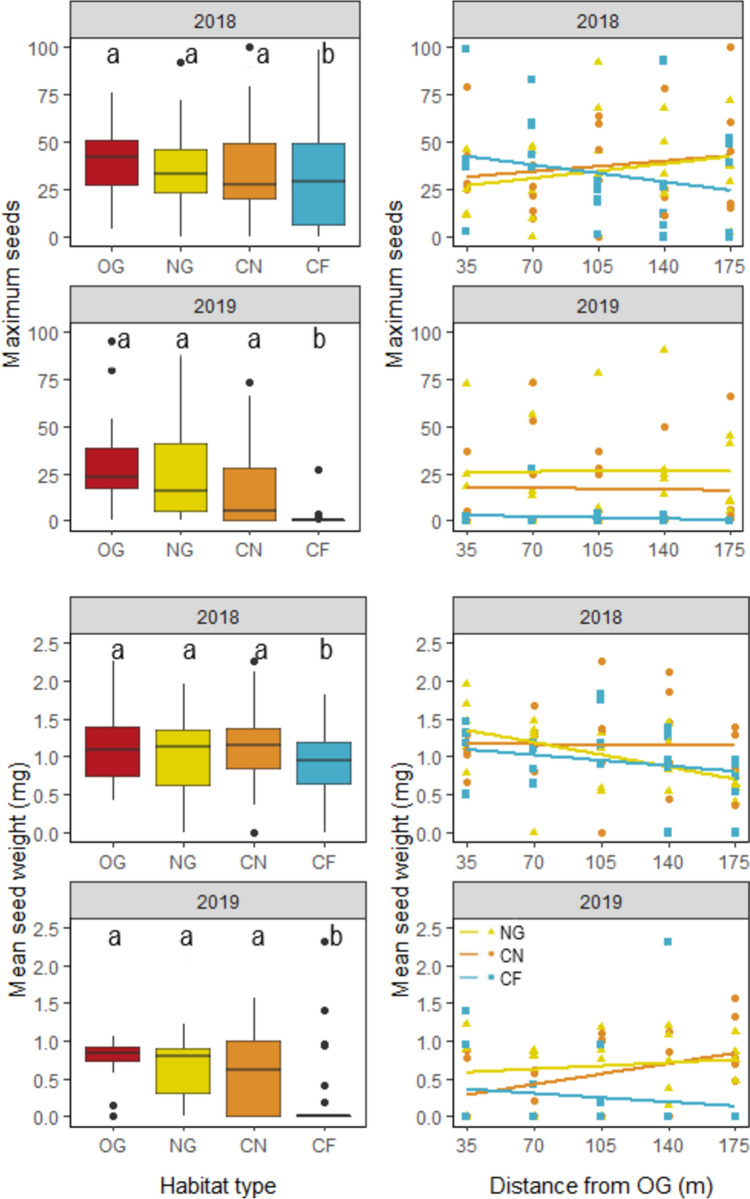
Pollination success—maximum number of *Hypochaeris radicata* seeds in one flower per study plot in the four habitat types (*OG* old grassland, *NG* new grassland, *CN* cereal near NG, *CF* cereal far from NG) in 2018 and 2019 (top left) and along the transects in NG, CN and CF with increasing distance from OG (top right). Mean seed weight (in mg) in the four habitat types in 2018 and 2019 (bottom left) and along the transects with increasing distance from OG (bottom right)

Significantly more seeds were produced in 2018 than in 2019. Over the 2 years, the number of maximum seeds was significantly lower in CF than in NG, OG and also in CN (Fig. 3; Table 2). The number of maximum seeds declined significantly within CF with increasing distance from OG. In contrast, there were no distance effects in CN and NG (Fig. 3; Table 2).

**Table 2:** Summary of GLMM and multicomparison Post hoc test testing for differences in maximum seeds between the four habitat types (*OG* old grassland, *NG* new grassland, *CN* cereal near, *CF* cereal far) and at different distances from OG within NG, CN and CF

Pollination success: maximum seeds—habitat types					
	Estimate	SE	*z* value	*p* value	
Intercept	3.66	0.25	0.00	< 0.001	***
OG	0.39	0.36	1.09	0.275	
CN	− 0.37	0.32	− 1.15	0.250	
CF	− 1.68	0.34	− 4.97	< 0.001	***
Y2019	− 1.78	0.25	− 7.13	< 0.001	***

**Multicomparison**					
*Contrast*	Estimate	SE	*z* ratio	*p* value	
NG—OG	− 0.39	0.36	− 1.09	0.695	
NG—CN	0.37	0.32	1.15	0.658	
NG—CF	1.68	0.34	4.97	< 0.001	***
OG—CN	0.76	0.36	2.09	0.157	
OG—CF	2.07	0.38	5.42	< 0.001	***
CN—CF	1.31	0.34	3.88	0.001	***

Pollination success:maximum seeds—distance effect					
	Estimate	SE	*z* value	*p* value	
Intercept	3.11	0.36	8.65	< 0.001	***
year2019	− 2.13	0.30	− 7.16	< 0.001	***
NG:Distance to OG	0.01	0.00	1.95	0.051	
CN:Distance to OG	0.00	0.00	0.85	0.397	
CF:Distance to OG	− 0.01	0.00	− 2.77	0.006	**

**Multicomparison**					
*Contrast*	Estimate	SE	*z* ratio	*p* value	
NG:Distance to OG—CN:Distance to OG	0.38	0.305	1.25	0.427	
NG:Distance to OG—CF:Distance to OG	1.72	0.333	5.16	< 0.001	***
CN:Distance to OG—CF:Distance to OG	1.34	0.334	4.01	< 0.001	***

Estimates of respective variable in the final model, standard errors (SE), *z*-values (z), *z*-ratios and *p*-values (*p*) with significant effects marked as . *p* < 0.1, *p < 0.05, **p < 0.01, ***p < 0.001

Mean seed weight was significantly lower in CF compared to the other three habitat types (Fig. 3; Table 3). Overall, mean seed weight declined significantly within CF with increasing distance from OG but not in CN or NG (Fig. 3; Table 3).

**Table 3:** Summary of LMM and multicomparison Post hoc test testing for differences in mean seed weight between the four habitat types (*OG* old grassland, *NG* new grassland, *CN* cereal near, *CF* cereal far) and at different distances from OG within NG, CN, and CF

	Estimate	SE	*t* value
**Pollination success: mean seed weight—habitat types**			
Intercept	1.14	0.08	13.953
OG	0.05	0.12	0.460
CN	− 0.03	0.10	− 0.250
CF	− 0.29	0.10	− 2.786
Y2019	− 0.50	0.07	− 6.791

Multicomparison					
Contrast	Estimate	SE	*z* ratio	*p* value	
NG—OG	− 0.05	0.12	− 0.46	0.968	
NG—CN	0.03	0.10	0.25	0.995	
NG—CF	0.29	0.10	2.79	0.027	*
OG—CN	0.08	0.12	0.68	0.906	
OG—CF	0.34	0.12	2.87	0.021	*
CN—CF	0.26	0.10	2.54	0.055	.

	Estimate	Std, Error	*t* value
**Pollination success: mean seed weight—distance effect**			
Intercept	1.064	0.112	9.547
NG:Distance to OG	0.001	0.001	0.502
CN:Distance to OG	0.001	0.001	0.750
CF:Distance to OG	− 0.002	0.001	− 1.834
Y2019	− 0.525	0.081	− 6.513

Multicomparison					
Contrast	Estimate	SE	*z* ratio	*p* value	
NG:Distance to OG—CN:Distance to OG	− 0.03	0.10	− 0.28	0.959	
NG:Distance to OG—CF:Distance to OG	0.26	0.10	2.61	0.024	*
CN:Distance to OG—CF:Distance to OG	0.28	0.10	2.89	0.011	*

Estimates of respective variable in the final model, standard errors (SE), *t*-values (t), *z*-ratios and *p*-values (*p*) with significant effects marked as . *p* < 0.1, *p < 0.05, **p < 0.01, ***p < 0.001

## Discussion

We could show that the establishment of NG within agricultural fields can enhance pollination services in CN. This confirms our second and third hypotheses regarding pollination and is in line with findings from previous studies reporting spill-over effects in arable fields adjacent to flower-rich semi-natural habitats (e.g., Williams et al. 2015). What we were not able to detect, however, was a spill-over effect for biological pest control within agricultural fields, which has been shown to be influenced by ecological contrasts, crop rotation and trophic levels of predatory groups (Boetzl et al. 2020a).

While other studies show a positive effect of flower strips and semi-natural habitats on biological pest control (Tschumi et al. 2015, 2016; Cahenzli et al. 2019; Albrecht et al. 2020), we were not able to observe this effect. This could be explained by inconsistent predator responses to NG within the study sites (Hussain et al. 2021). Species richness and activity density of carabids and ants did not increase in NG, at least not in the 3 years of observation, indicating slower adaptation of predatory insects to this new habitats. Rodenwald et al. (2023) found that flower strips could not outperform grassy field margins regarding biological pest control. In their study, both grassy field margins and flower strips enhanced the enemy-to-pest ratio for cereal leaf beetles but not for aphids. The parasitism rate for aphids was higher next to grassy strips than next to flower strips, giving altogether weak evidence for spill-over biocontrol effects from both semi-natural habitat types. They recommend protecting existing, permanent grassy strips as a cost-effective alternative to flower strips for promoting biocontrol services. Maas et al. (2021) showed that the differences in the spatio-temporal dispersal into NG between pollinators and predators are driven by species-specific functional traits. Key traits such as body size and hunting or foraging strategies seem to be the most defining factors for dispersal. Variation in dispersal rate affects the temporal mean and variability of ecosystem productivity through two mechanisms: spatial averaging by intermediate-type species that tend to dominate the landscape at high dispersal rates, and functional compensations between species that are made possible by the maintenance of species diversity (Loreau et al. 2003). While we found no difference in predation rate in OG and NG, species assemblages of predatory arthropods (carabids and spiders) constituted of different species in OG compared to NG and differences in species diversity were dependent on the taxonomic group (Hussain et al. 2021). We therefore assume that the community assembly time might be particularly important for ground-dwelling predator communities.

Assessing predation rates is intricate, and can rarely be linked directly to predator densities or functions. The prey card method is mainly used to assess the activity of ground-dwelling predators, which mostly do not depend on flower resources, unlike many flying predators and parasitoids. Boetzl et al. (2020b) tested the effectiveness of prey cards on the ground level as well as within the vegetation. Their use is especially recommended for assessments on the ground level and when time and resource limitations rule out more elaborate sentinel prey methods. In their study, predation rates on the ground level were three times higher than within the vegetation, which shows that this method might be less optimal to assess the predation efficiency of flying predators or those mostly found higher up in the vegetation. There are other methods for monitoring predation of flying arthropods such as video recording, the use of markers or the analysis of predators for prey remains, including molecular methods for analysis of predator gut contents (McCravy 2018). However, these methods are far more expensive and time-consuming, and their use on a larger scale is often difficult to implement. Nevertheless, it is important to consider the possibility that the inclusion of an additional monitoring technique for the predation rate of flying arthropods could potentially have shown a clearer effect for pest control in this study.

In Feng et al. (2021), species richness, abundance and Hill–Shannon diversity of carabids and linyphiids (Araneae: Linyphiidae) did not differ significantly between fallows and cereal fields and were not significantly related to the proportion of permanent grassland in the surrounding landscape. In contrast, the species composition of both communities differed significantly between cereal fields and fallows. Their results document considerable species turnover in natural enemy communities of adjacent arable fields and fallows, and support the assumption that only older fallows (> 8 years) produce functionally more diverse natural enemy communities. The most distinct differences between NG and OG are age (time after establishment) and consequently stage of plant succession, which might affect colonization rates of arthropods in NG (e.g., Kettermann et al. 2022; Gardiner and Casey 2022). However, in our study, we did not observe a difference in either pollination or biological pest control between NG and OG, effectively disproving our first hypothesis. This effect might, however, change with time.

While we were able to confirm a positive effect of NG on pollination within nearby crop fields, it is still a matter of discussion of how effective agri-environmental schemes are in promoting pollinators and pollination services. Zamorano et al. (2020) performed a meta-analysis which concluded that flower density of field margins correlates positively with the abundance and richness of pollinators at the field edge but has no consistent effect in the interior of the crop fields. The few studies that evaluated crop yield showed no effects, too. They conclude that field margin floral enhancements may constitute a positive conservation action for pollinators but are not necessarily associated with pollination ecosystem service. Scheper et al. (2023) show that biodiversity-friendly management on grasslands can significantly increase revenue on neighboring arable fields through positive effects on pollination service delivery. However, the opportunity costs of reduced grassland forage yields consistently exceeded the economic benefits of enhanced pollination. They conclude that profitability is often a key constraint hampering the adoption of biodiversity-friendly farming and that the actual implementation by farmers will largely depend on the availability of subsidies or other financial support from the public.

Another meta-analysis by Albrecht et al. (2020) observed that flower strips seem to indeed enhance pest control services in adjacent fields, however, effects on crop pollination and yield are more variable. Important drivers of this variability in effectiveness are an exponential decline in pollination services with distance from flower strips and age of plantings. Older flower strips with higher flowering plant diversity enhanced pollination more effectively. In our study, significantly more seeds were produced by the sentinel plants in 2018 compared to 2019. However, this most likely occurred due to better climatic conditions (higher temperatures and less precipitation) and therefore higher pollinator activity in 2018.

The effect of flower strips or semi-natural habitats on adjacent crop fields is also dependent on landscape context. Mota et al. (2022) conclude that in highly simplified agroecosystems such interventions may be insufficient or may need longer times to produce significant effects, yet, in regions where natural and semi-natural patches are already present, as was the case for our study region, the implementation of flower strips can be a successful strategy to promote pollinators and crop productivity.

Hussain et al. (2021) showed that bumblebees and solitary bees showed distinct distance decays in arable fields, however, syrphid (Diptera: Syrphidae) activity density declined in arable fields far away from the newly established grasslands, but not nearby. While pollinator foraging distances, especially of smaller bee species, have been underestimated in the past, the capability to use resources on a large spatial scale often applies only to a small percentage of individuals as some do not forage at distances longer than a few hundred meters. Generally, a close neighborhood of nesting and foraging habitat within a few hundred meters is important to maintain bee populations of smaller bee species (Zurbuchen et al. 2010). In our study, distances between plots and semi-natural habitats were generally well within common foraging distances of even small pollinators. We can therefore assume that pollinator mobility was not an issue and does not explain low pollination success in CF. Seed mixtures for NG consisted of two-thirds flowering plants and were cut only once a year. The particularly high density of pollen and nectar bearing plants was probably the determining factor for the high pollinator activity in NG with plants in CN benefitting from the proximity to NG. The significantly lower seed weight and the distance decay in seed numbers and seed weight in CF are additional indicators for the difference in pollination success between CN and CF. Excessive low seed weight and seed size can be a consequence of self-pollination and have been observed in several plant species (Craig 1989; Pellmyr et al. 1997; Cardoso 2004). Thus, we assume that low pollinator visitation rates in CF led to a higher self-pollination rate in the test plants and consequently to a lower mean seed weight.

Biodiversity loss and changes to functional composition are key mechanisms that underlie the impacts of land-use intensification on ecosystem-service delivery in managed grassland ecosystems (Allan et al. 2015). While some studies show that ecosystem services rely heavily on a few dominant pollinator species (Kleijn et al. 2015; Winfree et al. 2015), later results from the same authors indicate that many species are necessary to maintain ecosystem services in their entirety (Winfree et al. 2018). Enhancing key floral resources is essential to effectively mitigate the loss of pollinator diversity and associated provisioning of pollination functions in agro-ecosystems. Flower-rich NG seem to be an effective complement to OG to promote pollination services.

## Conclusion

We were able to confirm positive effects of new grasslands in nearby agricultural fields for pollination but not for biological pest control. Pollinators were shown to be clearly attracted by new grasslands (Hussain et al. 2021; Brandl et al. 2022), which resulted in increased pollination success on site and in adjacent arable fields. Conservation and restoration of existing semi-natural habitats needs to be of high priority, as they have already proven to be key for the biodiversity in agriculturally dominated areas (Plath et al. 2021; Shi et al. 2021; Šálek et al. 2022). Nevertheless, the establishment of permanent species-rich new grasslands should be used as a supplementary agri-environment measure, to enhance pollination and multifunctionality of grassland habitats in agricultural landscapes and contribute to the achievement of the EU Biodiversity Strategy for 2030 and the EU Pollinators Initiative to reverse the decline in wild pollinators by 2030.

### Supplementary Information

Below is the link to the electronic supplementary material.

Supplementary file1 (DOCX 26 KB)

## Data Availability

The datasets generated during and/or analysed during the current study are available from the corresponding author on reasonable request.
