# Supplementary material for: New grasslands promote pollination but not biological pest control in nearby arable fields in the short term
Source: Arthropod Plant Interact. 2024 Jan 31;18(2):327–38. doi: 10.1007/s11829-023-10034-5 (PMC10948462; doi:10.1007/s11829-023-10034-5)
Supplement: Supplementary file 1 — Supplementary file1 (DOCX 26 KB) [file 11829_2023_10034_MOESM1_ESM.docx]

# Supplementary Information

**New grasslands promote pollination but not biological pest control in nearby arable fields in the short term**

Journal: Arthropod-Plant Interactions

Manuela Bürgler | Raja Imran Hussain | Bea Maas | Ronnie Walcher | Dominik Rabl | Bernhard Krautzer | Dietmar Moser | Thomas Frank

**Correspondence**

Thomas Frank,

Institute of Zoology,

Department of Integrative Biology and Biodiversity Research,

University of Natural Resources and Life Sciences,

Gregor-Mendel-Straße 33, 1180 Vienna, Austria.

Email: [thomas.frank@boku.ac.at](mailto:thomas.frank@boku.ac.at)

Phone number: 0043 1 47654 83311

ORCID ID: 0000-0001-6377-719X

# Supplementary Information SI.1: Seed mixture and establishing success

**Table SI.1:** Overview of the plant species used for seed mixture in new grasslands (NG) and respective establishment success, sorted by plant type (grasses, herbs and legumes) and proportion in the seed mixture for NG, as well as the mean final coverage (in %) of each species in NG at the end of the three-year study (empty cells represent species that could not establish in NG). Final coverage was determined by an annual survey of the plant species present in the study plots.

| **Plant type** | **Species name** | **Proportion NG seed mixture (%)** | **Mean final coverage in NG (%)** |
| --- | --- | --- | --- |
| Grasses | *Arrhenatherum elatius* | 3.9 | 16.1 |
| Grasses | *Brachypodium pinnatum* | 1 |  |
| Grasses | *Briza media* | 1.9 | 0.0 |
| Grasses | *Bromus erectus* | 9.7 | 1.1 |
| Grasses | *Festuca ovina*agg. | 1 | 0.4 |
| Grasses | *Festuca rubra*agg. | 2.9 | 2.4 |
| Grasses | *Festuca rupicola* | 4.9 | 0.0 |
| Grasses | *Holcus lanatus* | 1 | 1.6 |
| Grasses | *Koeleria pyramidata* | 1.9 | 0.3 |
| Grasses | *Poa pratensis* agg. | 3.9 | 0.1 |
| Grasses | *Anthoxanthum odoratum* | 1.9 | 0.2 |
| Herbs | *Achillea millefolium* | 1 | 2.8 |
| Herbs | *Buphthalmum salicifolium* | 1.3 | 0.1 |
| Herbs | *Campanula patula* | 0.6 |  |
| Herbs | *Centaurea jacea* | 4.9 | 9.3 |
| Herbs | *Centaurea scabiosa* | 3.9 | 0.1 |
| Herbs | *Centaurea stoebe* | 0.6 | 1.2 |
| Herbs | *Crepis biennis* | 0.5 | 0.2 |
| Herbs | *Daucus carota* | 4.9 | 3.7 |
| Herbs | *Dianthus carthusianorum* | 1.9 | 0.4 |
| Herbs | *Galium mollugo* | 1.5 | 2.4 |
| Herbs | *Galium verum* | 1.9 | 0.2 |
| Herbs | *Hypericum perforatum* | 0.5 |  |
| Herbs | *Knautia arvensis* | 2.9 | 6.1 |
| Herbs | *Leontodon hispidus* | 1.9 | 0.3 |
| Herbs | *Leucanthemum vulgare* | 3.9 | 2.7 |
| Herbs | *Plantago lanceolata* | 3.9 | 0.7 |
| Herbs | *Plantago media* | 0.5 | 0.0 |
| Herbs | *Prunella grandiflora* | 1 | 0.0 |
| Herbs | *Ranunculus bulbosus* | 1 |  |
| Herbs | *Rumex acetosa* | 1 | 0.1 |
| Herbs | *Salvia pratensis* | 3.9 | 0.5 |
| Herbs | *Sanguisorba minor* | 1.9 | 0.6 |
| Herbs | *Silene nutans* | 1.9 |  |
| Herbs | *Silene vulgaris* | 3.9 | 1.2 |
| Legumes | *Anthyllis vulneraria* | 2.9 | 1.1 |
| Legumes | *Lotus corniculatus* | 2.9 | 6.6 |
| Legumes | *Medicago lupulina* | 1 | 2.4 |
| Legumes | *Onobrychis viciifolia* | 5.8 | 7.2 |
| Legumes | *Trifolium pratense* | 1 | 3.2 |
| Legumes | *Trifolium repens* | 1 | 4.1 |
| **Total coverage (%)** | | **100%** | **79%** |
